# Baclofen to prevent agitation in alcohol-addicted patients in the ICU: study protocol for a randomised controlled trial

**DOI:** 10.1186/s13063-016-1539-2

**Published:** 2016-08-19

**Authors:** Mickael Vourc’h, Fanny Feuillet, Pierre-Joachim Mahe, Véronique Sebille, Karim Asehnoune, The BACLOREA trial group

**Affiliations:** 1 Departments of Anaesthesiology and Surgical Intensive Care, Hôtel-Dieu, University Hospital of Nantes, 44093 Nantes, France; 2 Biometry Platform, Research Promotion Department, University Hospital of Nantes, 44093 Nantes, France; 3 EA 4275 SPHERE Methods for Patient-centered Outcomes and Health Research, Nantes University, 44035 Nantes, France

**Keywords:** Alcoholism, Intensive care, Baclofen, Restlessness, Delirium tremens, Alcohol withdrawal syndrome

## Abstract

**Background:**

Alcohol is the leading psychoactive substance consumed in France, with about 15 million regular consumers. The National institute on Alcohol Abuse and Alcoholism (NIAAA) considers alcohol abuse to be more than 14 units of alcohol a week for men and 7 units for women. The specific complication of alcoholism is the alcohol withdrawal syndrome. Its incidence reaches up to 30 % and its main complications are delirium tremens, restlessness, extended hospital stay, higher morbidity, and psychiatric and cognitive impairment. Without appropriate treatment, delirium tremens can lead to death in up to 50 % of patients.

**Methods/design:**

This prospective, double-blind, randomised controlled study versus placebo will be conducted in twelve French intensive care units (ICU). Patients with an alcohol intake level higher than the NIAAA threshold, who are under mechanical ventilation, will be included. The primary objective is to determine whether baclofen is more efficient than placebo in preventing restlessness-related side effects in the ICU. Secondary outcomes include mechanical ventilation duration, length of ICU stay, and cumulative doses of sedatives and painkillers received within 28 days of ICU admission. Restlessness-related side effects in the ICU are defined as unplanned extubation, medical disposal removal (such as urinary catheter, venous or arterial line or surgical drain), falling out of bed, ICU runaway (leaving ICU without physician's approval), immobilisation device removal, self-aggression or aggression towards medical staff. Daily doses of baclofen/placebo will be guided by daily creatinine clearance assessment.

**Discussion:**

Restlessness in alcoholic patients is a life-threatening issue in ICUs. BACLOREA is a randomised study assessing the capacity of baclofen to prevent agitation in mechanically ventilated patients. Enrolment of 314 patients will begin in June 2016 and is expected to end in October 2018.

**Trial registration:**

ClinicalTrials.gov Identifier: NCT02723383, registered on 3 March 2016.

**Electronic supplementary material:**

The online version of this article (doi:10.1186/s13063-016-1539-2) contains supplementary material, which is available to authorized users.

## Background

Alcohol is the leading psychoactive substance consumed in France with about 15 million regular consumers. The ESEMED4 study in 2001 [1] registered psychiatric disorders in about 10 % of men and 2.5 % of women with abusive alcohol intake. In 2013, alcohol was responsible for one death every 12 min in France [2]. In general hospitals, 20 to 35 % of men and 10 % of women suffer from chronic alcoholism [3]. In an intensive care unit (ICU), 28 % of admissions are related to abusive alcohol intake [4]. The National institute on Alcohol Abuse and Alcoholism (NIAAA) considers alcohol abuse to be more than 14 units of alcohol a week for men and 7 units for women.

ICU admission is an independent risk factor for delirium [5–7], pain-related health care issues, sleeping problems and neurological dysfunction [8–10]. The specific complication of alcoholism is alcohol withdrawal syndrome. Its incidence reaches up to 30 % [11] and its main complications are delirium tremens (DTs), restlessness, extended hospital stay, higher morbidity, and psychiatric and cognitive impairment [12–14]. Without appropriate treatment, DTs can lead to death in 50 % of patients [15]. Alcohol abuse leads to specific neurological dysfunction characterised by a fall in neuronal density [16] and consequently a higher risk of convulsion, confusion and restlessness [17]. Moreover, alcoholism also worsens vital prognosis [18, 19] and increases the morbidity and mortality of surgical patients [11, 20].

Alcohol withdrawal syndrome and delirium are commonly encountered in ICUs but standard treatment fails to reduce restlessness and related complications. Agitation related to DTs can lead to unplanned extubation and medical disposal removal and requires deep sedation, increasing the time on the ventilator. The beneficial effects of baclofen in preventing such a condition have never been reported in ICUs for at-risk drinkers.

Baclofen has been commercialised since 1974 and its target is the type-B γ-aminobutyric acid (GABA) receptor. In at-risk patients, this drug can reduce alcohol craving. Randomised studies have reported its efficiency in reducing both alcohol withdrawal syndrome [21, 22] and alcohol intake [23, 24] with conclusive safety data. Doses of up to 400 mg a day are commonly used, with very few side effects, in outpatients with working lives. Sixty to seventy percent of baclofen-treated patients reached total abstinence with a mean daily dose of 147 mg [25]. These data, taken altogether, have led the French medicinal and health products safety agency (ANSM) to advocate new randomised controlled trials to assess both the safety and efficiency of high doses.

Our objective is to take advantage of sedation and mechanical ventilation to allow clinicians to deliver high doses of baclofen without neuropsychiatric side effects for quick and marked effects, in an attempt to control restlessness, craving and related morbidity in ICU.

## Methods/design

### Design

BACLOREA is a prospective, multicentre, double-blind, randomised controlled study versus placebo, performed in at-risk drinkers according to NIAAA criteria. We will assess the superiority of baclofen compared to placebo in preventing restlessness-related complications. The randomisation sequence will be computer-generated and stratified at the 11 participating centres. This study follows the international recommendations for interventional trials (see the SPIRIT checklist in Additional file 1).

### Hypothesis

Early administration of baclofen to at-risk drinkers may reduce restlessness and its related complications when sedation is interrupted. We hypothesise that the administration of baclofen can reduce the incidence of patients who have at least one complication of agitation, including: unplanned extubation, medical disposal removal, falling out of bed, ICU runaway, immobilisation device removal, and self-aggression or aggression toward medical staff from 42 to 27 %.

### Trial intervention

After randomisation, the patients will receive double-blind baclofen or placebo. Daily doses will be adapted to daily modification of the diet in renal disease (MDRD) creatinine clearance from 150 to 50 mg (see Table 1). On the day of randomisation, the patient will receive the full daily dose in a one-shot administration. Then daily doses will be divided into three intakes on the following days. During the mechanical ventilation period, the treatment/placebo will be administered via the nasogastric feeding tube. After extubation, the treatment will be administered either via the nasogastric tube or the oral route. The doses were determined after a preliminary pilot study and previous trial analysis regarding efficiency and safety data [25].

**Table 1:** Doses of baclofen or placebo adjusted on daily assessment of the patient’s creatinine clearance

MDRD	D1 Loading dose	D2 and following days until extubation/tracheotomy at D15
Cl ≥90	150 mg	50-50-50
Cl 89–60	100 mg	30-20-50
Cl 30–59	70 mg	20-20-30
Cl <30 or continuous HD	50 mg	20-10-20
IHD	50 mg	50 mg before each session
Cl <15 without HD/IHD	50 mg	No administration

*Cl* renal clearance, *D* day, *HD* haemodialysis, *IHD* intermittent haemodialysis, *MDRD* modification of the diet in renal disease

The treatment will be tapered over a 7-day period after extubation or tracheotomy, or after the 15th day of treatment if the patient is still under mechanical ventilation. Treatment will be interrupted as described in Table 2.

**Table 2:** Gradual interruption of baclofen or placebo after extubation or tracheotomy or on the 15th day of treatment

Dose administered on the day of extubation/tracheotomy/15th day	150 mg	100 mg	70 mg	50 mg	IHD 50 mg
Day +1	100 mg (20-30-50)	70 mg (20-20-30)	50 mg (10-20-20)	30 mg (10-10-10)	30 mg before dialysis (no administration if the patient is not dialysed)
Day +2	70 mg (20-20-30)	50 mg (10-20-20)	30 mg (10-10-10)	20 mg (10-0-10)	30 mg before dialysis (no administration if the patient is not dialysed)
Day +3	50 mg (10-20-20)	30 mg (10-10-10)	20 mg (10-0-10)	10 mg (10-0-0)	10 mg before dialysis (no administration if the patient is not dialysed)
Day +4	30 mg (10-10-10)	20 mg (10-0-10)	10 mg (10-0-0)	Stop	Stop
Day +5	20 mg (10-0-10)	10 mg (10-0-0)	Stop		
Day +6	10 mg (10-0-)	Stop			
Day +7	Stop				

*IHD* intermittent haemodialysis

Clinical and biological safety data will be collected throughout the treatment period and the following 10 days, including blood count and liver function tests (see Additional file 2: SPIRIT Figure 3).

### Concomitant medication/treatment

All other interventions will be performed at the discretion of the attending physicians. Participating ICUs will monitor sedation and pain in ventilated patients using specific scales according to international guidelines [26].

### Population

At ICU admission, patients with a mechanical ventilation (MV) duration expected to be equal to or longer than 24 h may be included.

For patients with tracheal intubation at ICU admission:

- If intubation was performed less than 48 h before admission, inclusion can be performed within 24 h after admission
- If intubation was performed 48 h or more before admission, inclusion cannot be performed

For patients without tracheal intubation at ICU admission:

- If intubation was performed within the first 48 h after admission, inclusion can be performed in the first 24 h after intubation
- If intubation was performed at or after 48 h, inclusion cannot be performed

All patients, whatever the severity of organ failure, would be eligible for screening inclusion.

### Inclusion criteria

Adults aged from 18 to 70 years under mechanical ventilation for *1 day* or more with an estimated alcohol intake of:

- 14 units of alcohol per week during the month before hospitalisation for men aged 18 to 64
- 7 units of alcohol per week during the month before hospitalisation for women or men older than 65

### Exclusion criteria

Patients fulfilling one or more of the following criteria will not be included:

- Baclofen administration before ICU admission (personal treatment or single administration)
- Pregnancy
- Porphyria
- Burn injuries on ICU admission
- Personal treatment including gamma-hydroxybutyric acid (Alcover/Xyrem)
- Recent stroke or subarachnoid haemorrhage or head trauma with radiological evidence
- Paraplegia or tetraplegia
- Cardiac arrest with resuscitation manoeuvres before or after ICU admission
- Contraindication to enteral drug administration
- Lack of social protection
- Hypersensitivity to baclofen
- Coeliac disease
- Refractory epilepsy
- Dementia, schizophrenia, bipolar disorder or severe depression
- Parkinson’s disease
- Health care limitation owing to a poor prognosis
- Tracheotomy on ICU admission

### Randomisation and blinding

The clinicians will perform screening, enrolment and randomisation. Randomisation is centralised, web-based and accessible 24 h a day according to the allocation list on https://www.dirc-hugo-online.org/csonline/. Randomisation will be stratified at the centres using a fixed block size of 1:1 and a randomisation number will be allocated to each patient. Baclofen and placebo will be prepared and ‘blinded’ at Nantes University Hospital pharmacy. The independent trial statistician, patients, clinicians and nurses will be blinded for the allocation of treatment.

### Primary outcome measure

Composite score: occurrence (yes or no) of agitation-related adverse events during treatment/placebo administration with at least one sign out of the following:

- Unplanned extubation
- Medical disposal removal
- Falling out of bed
- ICU runaway
- Immobilisation device removal
- Self-aggression or aggression towards medical staff

### Secondary outcome measure

- Composite score: occurrence (yes or no) of agitation-related adverse events during treatment/placebo administration or 28-day mortality
- Adverse event (yes or no) related to agitation within 28 days of ICU admission
- Number of adverse events related to agitation occurring during ICU hospitalisation within 28 days of ICU admission
- Restlessness requiring rapid intravenous or intramuscular administration of hypnotic or neuroleptic drugs within 28 days of ICU admission
- Extubation failure defined as reintubation within the next 48 h
- Tracheotomy for failure of mechanical ventilation weaning
- Infections acquired in the ICU: urinary infection, pneumonia, catheter infection or bacteraemia
- Total doses of sedatives and painkillers received in the ICU within 28 days of ICU admission
- Riker Sedation Agitation Scale (SAS) in the ICU within 28 days of ICU admission
- Daily Clinical Institute Withdrawal Assessment of Alcohol Scale, revised (CIWA-Ar) alcohol withdrawal score during the week following extubation
- Duration of mechanical ventilation
- Ventilation-free days (VFD) at day 28
- Length of ICU stay
- Length of total hospitalisation
- Death in ICU, at days 28 and 90
- Death during hospital stay

### Participant withdrawal

Patients will be excluded from the trial if they or their next-of-kin withdraw their consent. As a result, the investigator will immediately interrupt treatment administration.

If the patient does not object to information on the primary outcome measure being obtained, the data will be collected and analysed. The data will be fully erased if the patient refuses this permission.

### Protocol suspension and severe adverse reactions

The treatment could be discontinued:

- Temporarily if:
  - ○ Renal clearance falls below 15 ml/min without haemodialysis support
  - ○ Liver tests (amino-transferase enzymes) are more than 20 times the normal
  - ○ Heart rate falls below 50 BPM with haemodynamic consequences
- Definitively if:
  - ○ Allergic symptoms appear
  - ○ Heart rate falls below 35 BPM whatever the haemodynamic profile
  - ○ Non-reactive uni/bilateral mydriasis develops
  - ○ Patient has a seizure in the ICU
  - ○ Patient has a stroke in the ICU with computed tomodensitometry (CT) scan confirmation
  - ○ Eyes not open 72 h after total sedation is interrupted

### Statistics

We hypothesised that baclofen administration can reduce the incidence of patients with at least one complication of agitation, using a two-sided test with an α of 5 % and 80 % power, from 42 to 27 %. This hypothesis in the placebo group is based on personal data. As a result, 314 patients are needed. Our study will include patients whatever their severity. Some patients may die before sedation interruption thus altering the power of the study. We therefore plan an interim analysis (after 50 % of inclusions), to adjust the sample size to maintain the statistical power. Friede and Kieser’s method [27, 28] allows both the initial hypothesis and the type-1 error planned a priori to be maintained. The analysis will be stratified at the participating centres. Statistical analysis will take place on an intention-to-treat principle. For the primary outcome, a logistic regression model will be applied to compare the proportion of agitation between the two treatment arms. Two sensitivity analyses will be performed and will consider dead patients to be without agitation, or as patients with or without agitation. A per-protocol analysis will also be performed to exclude patients with any of the exclusion criteria or without the inclusion criteria, or patients with incomplete treatment administration.

For the secondary outcomes, a Poisson regression model will be applied to compare the number of adverse events related to agitation, between the two treatment arms. Both length of ICU stay and mechanical ventilation will be compared with a log-rank test. Logistic regression will be used to compare binary qualitative secondary endpoints.

Normally distributed variables will be expressed as mean and standard deviation; nonnormally distributed variables will be expressed as median and interquartile range. Categorical variables will be expressed as numbers and percentages.

### Track record

Data will be recorded in the web-based electronic case report form (eCRF) by trial site staff. Characteristics at baseline will be gathered: age, site of inclusion, sex, weight, height, medical history, reason for ICU admission, alcohol consumption, kidney function, Simplified Gravity Index (IGSII) and Sequential Organ Failure Assessment (SOFA) scores on admission. During ICU hospitalisation we will assess: cumulative doses of all sedative drugs, agitation and alcohol withdrawal scores, agitation-related adverse events, blood tests results regarding alcohol test on admission, liver function tests and blood count during treatment, acquired infection, length of ICU stay, mechanical ventilation and whole hospitalisation. To preserve confidentiality of participant’s personal information, data will be key-coded using alphanumerical numbers. To minimise missing data, primary and secondary outcome will be notified daily. Patients will be monitored until day 90 and a supplemental telephonic contact will promote complete follow-up.

### Monitoring

Monitoring will follow Good Clinical Practice principles and will be performed by the independent promotion department of Nantes University Hospital Research Management Unit. A clinical research associate (CRA) will visit each centre every 6 months to control the quality of the recorded data. The CRA will be able to check the case report forms and medical records. The following data will be assessed:

- Written informed consent
- Flow chart filled in for included and excluded patients
- Trial progress
- Primary and secondary outcome collection
- Treatment-related severe adverse events
- Stock management of the study treatment

Participating centres agree to accept the promoter’s quality audits.

### Trial status

Patients from 11 ICUs are expected to be included over a 2-year inclusion period. We plan to include two patients per ICU per month.

2015: protocol approval by the Ethics Committee and French national drug safety agency.

June 2016–2018: inclusions

Beginning of 2017: interim analysis

2018: closing of the database. We will submit our manuscript during the second half of 2018.

## Discussion

Restlessness in alcoholic patients is a life-threatening issue in ICUs. Baclofen usefullness was already reported to treat alcohol withdrawal syndrome, that's why its accuracy in ICU had to be assessed by large multicentre studies. Invasive Mechanical ventilation and continuous tight monitoring will allowed to introduce relatively high doses of Baclofen to reach fast effective plasma drug concentration and ensure patient security. In parallel, reducing agitation may avoid other hypnotic drugs administration and so reduce their side effects: Mechanical ventilation duration and its complications, delirium and neuro-psychiatric aftereffects.

## Additional files

Additional file 1: SPIRIT checklist. (DOC 117 kb) Additional file 2: Figure 3 Study schedule. Caption of Figure 3: ICU, Intensive care unit; SAS, Riker Sedation Agitation Scale; CIWA-Ar, Clinical Institute Withdrawal Assessment of Alcohol Scale, revised; NIAAA, National Institute on Alcohol Abuse and Alcoholism. (DOCX 136 kb)

## Abbreviations

ANSM: ANSM French medicinal and health products safety agency
CIWA-Ar: Clinical Institute Withdrawal Assessment of Alcohol Scale, revised
Cl: Clearance
CRA: Clinical research associate
CT: Computed tomodensitometry
CVVHD: Continuous veno-venous haemodialysis
DSMB: Data and Safety Monitoring Board
DTs: Delirium tremens
eCRF: Electronic case report form
ESEMED: European Study of the Epidemiology of Mental Disorders
IHD: Intermittent haemodialysis
ICU: Intensive care unit
IGSII: Simplified Gravity Index
MDRD: modification of diet in renal disease
NIAAA: National Institute on Alcohol Abuse and Alcoholism
SAS: Riker Sedation Agitation Scale
SOFA: Sequential Organ Failure Assessment
VFD: Ventilation-free days
