# Supplementary material for: Baclofen to prevent agitation in alcohol-addicted patients in the ICU: study protocol for a randomised controlled trial
Source: Trials. 2016 Aug 19;17:415. doi: 10.1186/s13063-016-1539-2 (PMC4992221; doi:10.1186/s13063-016-1539-2)
Supplement: Supplementary file 2 [file 13063_2016_1539_MOESM2_ESM.docx]

	ICU stay					
	INCLUSION D1	Sedation time	Tube removal/trachéo	D28 (or ICU release < D28)	ICU release	D90 follow up
Screening of inclusion/exclusion criteria	X					
Patient or next-of-kin information	X					
Consent (Patient/next-of-kin/emergency)	X					
Randomisation	X					
Pregnancy test for all women < 45 years old.	X					
**TREATMENT ADMINISTRATION (ENTERAL ROUTE) = 7.00 - 15.00 - 23.00**	X<br>(1 shoot)	**Daily administration until tube removal or tracheotomy followed by a 7-day decrease.<br>If the patient is not weaned from mechanical ventilation, total treatment duration must not exceed 15 days followed by a 7-day decrease (total of 22 days).**				
Blood count, Renal functionand glycemia assessement once a day	X	**As long as the treatment is administered**				
Liver test once a week	X	**As long as the treatment is administered**				
**PRIMARY OUTCOME**						
Unplanned extubation<br>Medical disposal removal<br>Falling out of bed<br>ICU runaway<br>Immobilization device removal<br>Self aggression or toward medical staff.		**As long as the treatment is administered<br>(Primary outcome is recorded as long as treatment is administered, but all adverse events with day 28 after ICU admission have to be recorded)**				
**SECONDARY OUTCOMES**						
ICU-acquired infection		**Until ICU release**				
Total hypnotic doses administered in ICU		**From day 1 to day 28**				
Worst SAS Score in 24-hour		**From day 1 to day 28**				
CIWA-Ar alcohol withdrawal score (once a day at 8.00 AM)			**During the 7 days following tube removal or tracheotomy**			
Morbidity and Mortality in ICU		**Until ICU release**				**X**
NIAAA alcohol abuse questionnaire					**X**	
